# H_2_S in acute lung injury: a therapeutic dead end(?)

**DOI:** 10.1186/s40635-020-00324-0

**Published:** 2020-12-18

**Authors:** Tamara Merz, Nicole Denoix, Martin Wepler, Holger Gäßler, David A. C. Messerer, Clair Hartmann, Thomas Datzmann, Peter Radermacher, Oscar McCook

**Affiliations:** 1 Institute for Anesthesiological Pathophysiology and Process Engineering, Ulm University Medical Center, Helmholzstraße 8/1, 89081 Ulm, Germany; 2 Clinic for Psychosomatic Medicine and Psychotherapy, Ulm University Medical Center, Ulm, Germany; 3 Clinic for Anesthesia, Ulm University Medical Center, Ulm, Germany; 4 Clinic for Anesthesia and Intensive Care, Bundeswehrkrankenhaus, Ulm, Germany

**Keywords:** Suspended animation, Gaseous mediator, Hypometabolism, Inflammation, Oxidative stress, Translational medicine

## Abstract

This review addresses the plausibility of hydrogen sulfide (H_2_S) therapy for acute lung injury (ALI) and circulatory shock, by contrasting the promising preclinical results to the present clinical reality. The review discusses how the narrow therapeutic window and width, and potentially toxic effects, the route, dosing, and timing of administration all have to be balanced out very carefully. The development of standardized methods to determine in vitro and in vivo H_2_S concentrations, and the pharmacokinetics and pharmacodynamics of H_2_S-releasing compounds is a necessity to facilitate the safety of H_2_S-based therapies. We suggest the potential of exploiting already clinically approved compounds, which are known or unknown H_2_S donors, as a surrogate strategy.

## Background

This review explores the plausibility of hydrogen sulfide (H_2_S) therapy for acute lung injury (ALI) and circulatory shock. H_2_S is a toxic gas with a characteristic smell of rotten eggs, and is also produced endogenously by three different enzymes: cystathionine-β-synthase (CBS), cystathionine-γ-lyase (CSE), and 3-mercaptopyruvate-sulfurtransferase (MST) [1]. In 1996 and 1997, physiological roles of H_2_S in the brain and vascular smooth muscle, respectively [2, 3], were discovered, which led to its classification as the third “endogenous gaso-transmitter” [4], besides nitric oxide and carbon monoxide.

In 2005, in a hallmark study, Blackstone et al. demonstrated that inhaled H_2_S (80 ppm, ambient temperature 13 °C) can induce a “suspended-animation” like state by reduction of the metabolic rate in spontaneously breathing mice. This was accompanied by a fall in body temperature down to 15 °C [5]. The metabolic rate dropped by 90% after 6 h of H_2_S exposure. The effect was fully reversible upon transferring the mice into room air and room temperature [5]. These findings led to high hopes and a frenzy of speculation regarding the ability of H_2_S to induce a hypometabolic state which could be exploited in patient care [6]. However, the fact that this effect was first shown in experimental conditions (low ambient temperature, no maintenance of body temperature, and no anesthesia) that are contrary to the current clinical practice, some drawbacks have to be anticipated in translating this effect to critical care medicine. Interestingly, H_2_S-induced hypometabolism and hypothermia could be reproduced in mice at room temperature, but could not be confirmed in anesthetized sheep [7]. In anesthetized pigs, Simon et al. did report a sulfide-induced drop in metabolism in a model of aortic occlusion with intravenous sulfide administration [8]. However, in large animals, the effect seems to take longer to manifest and is not as pronounced as in rodents. Thus, the concept of H_2_S-induced “suspended animation” or hypometabolism should remain in the realm of science fiction (as suggested by Drabek et al. [9]), but it is also true that potentially therapeutic effects of H_2_S independent of hypometabolism [10–12]: anti-inflammatory, antioxidant, organ-specific benefits, regulation of blood pressure, and glucose metabolism [13–17], are encouraging for the clinical development of H_2_S donors and have not yet been fully explored [18]. After a brief introduction into the role of H_2_S in the lung, its role in chronic lung diseases and modes of exogenous H_2_S administration, we will review the current literature of exogenous H_2_S administration in preclinical models of acute lung injury (ALI, mostly rodents), translationally more relevant models of lung injury and circulatory shock (resuscitated large animal models), and finally conclude with the current status of clinical trials of H_2_S therapies and an outlook on future clinical development.

### The role of H_2_S in the lung

High levels of H_2_S gas have been shown to be an environmental hazard, entering the body through the lung and being further distributed via the bloodstream [17]. H_2_S as a byproduct of various industries and pollutant arising from sewers can cause a “knock-down” effect upon inhalation of > 500 ppm: pulmonary injury, loss of consciousness, cardiopulmonary arrest, and death [19]. Generally, 10–20 ppm of H_2_S are considered to be safe to inhale acutely [17]. The effects of a chronic low-level exposure to H_2_S on lung toxicity have not been well characterized, and epidemiological studies are controversial, either reporting no relevant effect [20], or reduced lung function [21]. Bates et al. investigated the effects of naturally occurring H_2_S in geothermal areas on pulmonary health and found no detrimental effect and surprisingly even suggest a potential benefit on lung function [22].

H_2_S reportedly plays a role in lung development [23], and a deficiency in the endogenous H_2_S enzymes impairs alveolarization [24]. In the adult lung, the expression of the endogenous enzymes has been identified in a variety of pulmonary compartments in different species: rodents [25–27], bovine [28], and humans [29–32]. An upregulation of the endogenous H_2_S enzymes has been reported to play a role in the adaptive response to injury [27, 33]. However, the role of endogenous H_2_S in the adult lung is not well established.

### H_2_S in chronic lung diseases

Chronic pulmonary diseases have been found to be associated with reduced H_2_S serum levels in patients [34] and suppressed pulmonary CSE expression [31]. Even though a few preclinical studies report pro-inflammatory effects of H_2_S in general (e.g., [35, 36]), it seems well established that the predominant H_2_S effect in the pathophysiology of chronic pulmonary diseases is anti-inflammatory [25, 31, 32, 37, 38]. Interestingly, low expression of the H_2_S-producing enzymes was shown to compromise the anti-inflammatory effects of glucocorticoid therapy in asthma [31, 39]. Low levels of CSE expression and H_2_S production in early development have been correlated to a higher susceptibility to allergic asthma in young mice [40]. The protective role of H_2_S in chronic inflammatory lung diseases has been thoroughly reviewed by Chen and Wang ([41]: animal models [25, 37, 39, 42] and human studies [34, 43]) and reported more recently (animal models: [38, 44] human: [31], human in vitro: [32]). There are numerous studies reporting a potential benefit of exogenous H_2_S administration in chronic lung diseases [25, 32, 38, 44, 45].

### Possible strategies for exogenous administration of H_2_S

The possible strategies for exogenous administration of H_2_S have been reviewed recently by Szabo and Papapetropoulos [17] and comprise the following: inhalation of gaseous H_2_S and intraperitoneal (i.p.) or intravenous (i.v.) administration of various H_2_S-releasing compounds: H_2_S-releasing salts (e.g., Na_2_S, NaHS) and slow H_2_S-releasing donors (GYY4137, AP39, diallyl-trisulfide (DATS)). Regarding the effects of exogenous H_2_S on inflammation reveals that short-term free sulfide levels as a consequence of the administration of H_2_S-releasing salts can have detrimental effects, whereas a slow continuous H_2_S release from slow-releasing donors attenuated inflammation (demonstrated in vitro by [46] and thoroughly reviewed by [13]). An overview of currently available H_2_S-releasing compounds is given in Table 1.

**Table 1:** Overview of various sulfide donors and their sulfide release

Donor category	Compounds	Sulfide release
**Inhalation**	Gaseous H_2_S	Rapid, high risk of toxic peak concentrations
**Sulfide-releasing salts**	Na_2_S, NaHS, IK-1001	Rapid, high risk of toxic peak concentrations
**Slow-releasing donors**	GYY4137, AP39, DATS, SG-1002	Slow, toxicity ultimately not clear
**Clinically available compounds**	Sodium thiosulfate (STS), Ammonium tetrathiomolybdate (ATTM), Zofenopril	Slow, good safety profile

## Therapeutic potential of H_2_S during acute lung injury

In the following subsections, 70 articles investigating the effects of exogenous H_2_S administration in various models of acute lung injury are reviewed. These articles were identified in a literature search on PubMed in August 2019 with the search term “hydrogen sulfide” in combination with either “acute lung injury” or “ventilator-induced lung injury” or “shock” and “lung.” Articles that were not available in English or did not deal with exogenous H_2_S administration were excluded.

### Ventilator-induced lung injury (VILI)

The effects of exogenous H_2_S in murine models of VILI are mostly reported to be anti-inflammatory. Only one study reports an acceleration of VILI with 60 ppm of H_2_S gas administration as an inhaled gas [47]. However, in the same study, pre-treatment with an intra-arterial bolus of Na_2_S (0.55 mg/kg) before starting harmful ventilation could attenuate lung inflammation and oxidative stress [47]. The latter is well in accordance with the protective effects of H_2_S in VILI reported by Aslami et al. and Wang et al., who observed reduced inflammation and improved lung function in animals with VILI, treated with a continuous infusion of 2 mg/kg/h NaHS or DATS, respectively [48, 49]. In contrast to the harmful effects of gaseous H_2_S administration (60 ppm) [47], four separate reports from a different group all indicate a beneficial effect of 80 ppm of H_2_S: anti-inflammatory and anti-apoptotic effects [11], attenuated lung damage [50], antioxidant effects [51], and prevention of edema formation, even with a reduced H_2_S administration time [52]. These contrasting results might be due to the fact that the latter group used a milder VILI protocol with a tidal volume of 12 ml/kg over a longer time (6 h) [11, 50–52] rather than 40 ml/kg for 4 h as [47]. In conclusion, these results suggest an overall beneficial effect of H_2_S in VILI.

### Pancreatitis-induced acute lung injury (ALI)

Up to 1/3 of all pancreatitis patients develop ALI or acute respiratory distress syndrome (ARDS), which accounts for 60% of pancreatitis-related deaths [53]. Inhibition of cystathionine-γ-lyase (CSE) had anti-inflammatory effects in a murine model of pancreatitis-induced lung injury [54]. In a follow-up experiment, Bhatia et al. 2006 reported an induction of lung inflammation and histological damage in response to i.p. injection of 10 mg/kg NaHS in mice [55]. The effects were only present 1 h post-injection and by 3 and 6 h, the inflammatory state had returned to baseline [55], suggesting that the toxic effects were a transitory consequence of NaHS-induced high peak sulfide concentrations, which were quickly cleared. Besides Bhatia et al. 2005 [54], three more studies report a benefit of the inhibition of endogenous H_2_S production by CSE (either chemically or genetic deletion) on pancreatitis-induced ALI in murine models [56–58]. However, as mentioned previously, the effects of H_2_S on inflammation are controversial: in other studies, both the administration of ACS15 (H_2_S-releasing diclofenac) and NaHS pre-treatment (10–15 mg/kg) led to an attenuation of inflammation in pancreatitis-induced ALI [59, 60]. The context of H_2_S administration seems to be crucial: in a healthy animal, 10 mg/kg NaHS induces transient lung inflammation, whereas this kind of pre-treatment is anti-inflammatory in subsequent pancreatitis-induced ALI. Furthermore, the role of CBS in the CSE inhibition experiments is not clear—it could potentially be upregulated in response to CSE inhibition. Neither of the CSE inhibition experiments report pulmonary H_2_S levels; thus, no causal conclusions about the role of H_2_S itself in inflammation can be drawn from these studies.

### Burn and/or smoke-induced lung injury

Acute lung injury is common in burn injury patients and can also be aggravated by the inhalation of smoke. In a murine model of hot water-induced skin burn, Zhang et al. observed aggravated lung inflammation and histological damage in animals treated with NaHS (10 mg/kg) [61], which could be mediated by transient toxic peak sulfide release, which has to be anticipated with this dose of NaHS. In contrast, in a similar model, Ahmad et al. report attenuated pulmonary cell infiltration and oxidative stress with the administration of AP39 [62]. However, confoundedly, another arm in this study was treated with AOAA, an inhibitor of endogenous H_2_S enzymes [63], which had the same effects as AP39, prompting their conclusion of a “complex pathogenic role of H_2_S in burns” [62]. However, the authors neither report H_2_S levels nor the expression levels of the endogenous enzymes, which makes it difficult to interpret their data. In the lung, the upregulation of the endogenous H_2_S enzymes can represent an adaptive response to stress [27]. Thus, it is tempting to speculate that their apparently ambivalent results may be attributed to AOAA and AP39 having a similar regulatory effect on the endogenous H_2_S enzymes, which has not been investigated or reported yet. In fact, Han et al. report attenuated lung injury and antioxidant effects of spontaneous breathing of 80 ppm H_2_S in a rat model of cotton smoke-induced ALI [64]. In a combined model of smoke- and flame burn-induced lung injury, Esechie et al. were able to demonstrate attenuated inflammation and improved 5 days survival due to subcutaneous Na_2_S treatment [65]. They were also able to confirm this protective effect of Na_2_S in a large animal (ovine) model of smoke and burn injury, where a 24-h primed continuous i.v. infusion of Na_2_S after injury ameliorated pulmonary pathophysiological changes [66]. Overall, H_2_S seems to mediate protective effects in burn- and/or smoke-induced ALI.

### Endotoxin-induced ALI

All studies investigating the effects of exogenous H_2_S in LPS-induced lung inflammation were performed in rodents and reported beneficial effects, regardless of the mode of LPS (locally or systemically) and H_2_S (salt, slow-releasing donor, inhalation) administration. Inhalation of 80 ppm H_2_S after intranasal LPS attenuated lung histological damage and had anti-inflammatory and antioxidative effects [67, 68]. Pre-treatment with GYY4137 also attenuated lung injury and cell infiltration after LPS inhalation [69]. Both GYY4137 and NaHS pre-treatment also attenuated lung injury and inflammation after intratracheal LPS exposure [70, 71]. A therapeutic administration of H_2_S, either sodium thiosulfate (STS) or GYY4137, after intratracheal LPS ameliorated pulmonary inflammation as well [72, 73]. GYY4137 also attenuated cell infiltration in the lung after i.v. injection with LPS. Pre-treatment with GYY4137 had antioxidant and anti-inflammatory effects in i.p. injection of LPS. NaHS administration 3 h after i.v. LPS attenuated inflammation and oxidative stress and protected the mitochondria in the lung [74].

### Polymicrobial sepsis-induced ALI

In contrast to studies investigating endotoxin administration, the role of exogenous H_2_S in murine models of cecal ligation and puncture (CLP, abdominal sepsis) is controversial: both beneficial and detrimental effects have been reported. In a resuscitated murine model, 100 ppm of inhaled H_2_S had minor anti-inflammatory effects, though not mediating protective effects in CLP [75]. A variety of studies report aggravation of sepsis-induced lung injury by NaHS [76–82]. However, in all these models, NaHS was administered as an i.p. bolus and did not comprise any additional resuscitative measures. The route of administration might also be a confounding factor combined with the CLP. Furthermore, the dose of H_2_S that was used in these studies was much higher than the dose of the previously mentioned LPS experiments (i.e., 10 mg/kg during CLP versus 0.78–3.12 mg/kg i.p. NaHS during LPS). In fact, 1 h i.v. administration of NaHS at a rate of 1 and 3 mg/(kg × h) after CLP attenuated oxidative stress and cell infiltration in the lung [83]. High peak sulfide levels achieved by the bolus administration of a high dose of H_2_S can exert toxic detrimental effects, whereas achieving a less pronounced elevation of sulfide levels over a longer period of time could exert a benefit [13]. In a model of enterocolitis, the slow-releasing H_2_S donor GYY4137 attenuated lung inflammation and edema, whereas Na_2_S (20 mg/kg 3 times daily) had no effect [84].

### Oleic acid-induced ALI

ALI is most commonly modeled in mice by an intravenous injection of oleic acid (OA) [85]. Studies investigating exogenous H_2_S administration in this model consistently report beneficial effects: attenuated edema formation, reduced cell infiltration, and anti-inflammatory and antioxidant effects of NaHS pre-treatment [86–89].

### Oxidative lung injury

In models of hyperoxia- or ozone-induced ALI, NaHS administration exerted anti-inflammatory and antioxidative effects [90–92]. However, hyperoxia cannot only induce lung damage, depending on the experimental protocol: hyperoxia, as an experimental therapy in combined fracture healing and blunt chest trauma, exerted lung-protective effects. Interestingly, these protective effects were associated with an amelioration of the stress-induced upregulation of endogenous H_2_S enzymes and thus restoring the naive state of protein expression [27].

### Trauma-induced ALI

Blunt chest trauma induces mechanical and inflammatory injury to the lung [93]. In a resuscitated, murine model of thoracic trauma, a continuous i.v. infusion of Na_2_S (0.2 mg/(kg × h)) had no effect on lung mechanics and gas exchange, but reduced apoptosis and cytokine production [33]. These effects were even more pronounced in combination with hypothermia [33]. Inhaled H_2_S (100 ppm) attenuated inflammation and cell infiltration in the lung in a non-resuscitated rat model of thoracic trauma [94]. However, in both these studies, the effects of H_2_S were rather weak and a clear benefit could not have been determined [33, 94], in contrast to models of other types of injury. Interestingly, an upregulation of pulmonary CSE expression in response to combined acute on chronic lung disease, i.e., thoracic trauma after cigarette smoke exposure, was suggested to be an adaptive response to injury [27, 95], in that a genetic deletion of CSE in the same kind of acute on chronic trauma was associated with aggravated ALI [96].

### ALI in various types of ischemia/reperfusion injury (I/R)

In a rat model of lung transplantation, NaHS (0.7 mg/kg i.p.) improved lung function and reduced cell infiltration and oxidative stress [97]. NaHS pre-treatment was beneficial in limb I/R-induced lung injury, due to anti-inflammatory effects and attenuated edema formation [98]. GYY4137 pre-treatment has been tested in infrarenal aortic cross clamping, as well as lung I/R, and beneficial effects have been reported in both types of lung injury: anti-inflammatory and antioxidant activity, respectively [99, 100]. Results in models of hemorrhagic shock are controversial. One study found a beneficial effect of an i.p. bolus of NaHS in a rat model: attenuated edema formation, cell infiltration, and necrosis [101]. Another study of HS in mice determined pulmonary anti-inflammatory effects of AP39; however, the mortality rate in the treated arm of this study was very high due to profound vasodilation [102]. Using a lower dose of AP39 yielded no effects at all [102]. These opposite effects of exogenous H_2_S administration in these two experiments might be due to the different H_2_S-releasing compounds used or resuscitative measures. Chai et al. [101] performed the re-transfusion/resuscitation only with fluid administration, whereas Wepler et al. [102] used re-transfusion of shed blood and a full-scale small animal intensive care unit (ICU) setup (see below), which certainly changes the pathophysiology. In general, the role of H_2_S in hemorrhagic shock is controversial, with either a beneficial [103–108], harmful [109, 110], or no impact [111, 112].

## Translational medicine—H_2_S in large animal models of shock

Animal models with the purpose to identify relevant novel therapeutic strategies for patient care should reflect the clinical situation as closely as possible. In the context of ALI and shock research, the clinical practice for patient care in the ICU has to be reflected in experimental models to facilitate the translation from preclinical research to the clinical reality, i.e., temperature management, frequent blood gas analysis, lung-protective mechanical ventilation, hemodynamic monitoring, fluid administration, and catecholamine support titrated to the mean arterial pressure (MAP) [113]. Metabolic and organ-specific differences between small and large animals need to be taken into account [114, 115], as well as the challenge of reproducing the patient’s pathophysiology (e.g., comorbidities and premedication).

In particular for H_2_S, in a translational scenario, the implementation of intensive care measures (e.g., maintenance of body temperature, anesthesia, fluid resuscitation) might interfere with its effects, thus contributing to the lack of a hypometabolic effect in resuscitated rodent intensive care models [10, 33, 75, 102]. In large animals, the effects of H_2_S administration, in general, have been less robust, not only due to the intensive care measures, but also due to their large body size and different metabolic and thermoregulatory phenotype [114]. Large resuscitated animal studies reflect (i) no or very limited effects [8, 103, 112, 116–118], (ii) organ-specific effects [66], or (iii) beneficial effects restricted to a narrow timing and dosing window [119, 120].

As aforementioned, the induction of suspended animation by H_2_S inhalation was successful in small animals [5]; however, the translation to larger animals and eventually humans has proven to be challenging. Small animals have a much higher metabolic rate in relation to their body weight than large animals [121]; thus, the induction of a hypometabolic state is much easier to perform in small animals [114]. To induce that same state in a larger animal, a much higher dose of H_2_S would be needed, harboring the risk of toxicity [114]. However, the challenges of measuring H_2_S/sulfide in biological samples make it difficult to perform dose-finding studies.

Nonetheless, several studies in large animal models explored the therapeutic potential in various types of ALI. Na_2_S in an ovine model of burn reduced mortality and improved gas exchange [66]. In porcine models, Na_2_S was further studied in hemorrhagic shock, where it attenuated lung damage when administered at the time of reperfusion, however largely unrelated to hypothermia [120]. Administration of STS in the acute phase of resuscitation (24 h) after hemorrhagic shock in a porcine comorbid atherosclerotic model showed only a limited effect by improved gas exchange and lung mechanics in comparison to vehicle-treated animals (Table 2, [122]). Nußbaum et al. investigated the effects of GYY4137 during long-term resuscitated septic shock in pigs with atherosclerosis: GYY4137 treatment led to a preferential utilization of carbohydrates; however, they did not observe any major benefit of the treatment, gas exchange was not affected, and they did not further investigate lung tissue [117]. Unfortunately, none of the other large animal studies report lung function or lung histopathology. Still, it seems that exogenous H_2_S can mediate lung-protective effects in translationally relevant large animal models, when carefully timed and titrated.

**Table 2:** Lung function in a resuscitated comorbid porcine model of hemorrhagic shock [122]

Timepoint	Group assignment	Horowitz index (mmHg)	PEEP (cmH_2_O)
Baseline	Control	400 (338, 448)	0
	Thiosulfate	351 (328, 427)	0
After shock (start of STS infusion)	Control	376 (322, 431)	0
	Thiosulfate	352 (283, 405)	0
24 h after shock (end of STS infusion)	Control	387 (326, 418)	10 (10, 10)
	Thiosulfate	385 (355, 417)	10 (10, 10)
48 h after shock	Control	230 (195, 270)#	12.5 (12.5, 15)
	Thiosulfate	299 (263, 339)*	11.3 (10, 12.5)
72 h after shock	Control	289 (106, 323)#	15 (12.5, 15)
	Thiosulfate	337 (300, 387)	10 (10, 12.5)*

Atherosclerotic pigs were surgically instrumented and, after a short recovery period, underwent 3 h of hemorrhagic shock (target mean arterial pressure 40 ± 5 mmHg). Seventy-two hours of resuscitation comprised re-transfusion of the shed blood and fluid and catecholamine administration targeted to the pre-shock mean arterial pressure. Further details about the experimental protocol can be found in [122]. STS was administered during the first 24 h of resuscitation after hemorrhagic shock. Effects on lung function were most pronounced at 48 h after hemorrhagic shock. Data shown are median (lower quartile, upper quartile)

*Significant to control group

#Significant to baseline (*p* < 0.05 in two-way ANOVA)

## Clinical trials of exogenous H_2_S administration in ALI

**Fig. 1:**
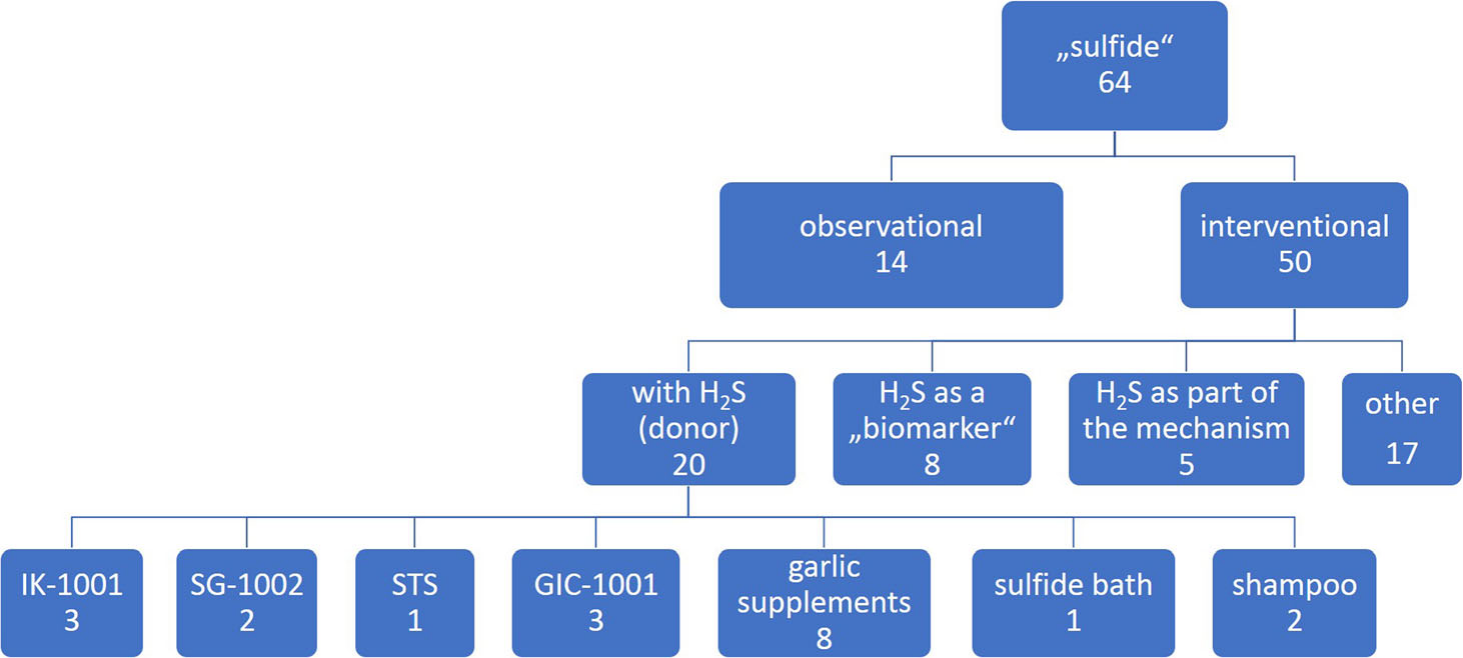
Overview of clinical trials found with the search term “sulfide” listed on clinicaltrials.gov

**Fig. 2:**
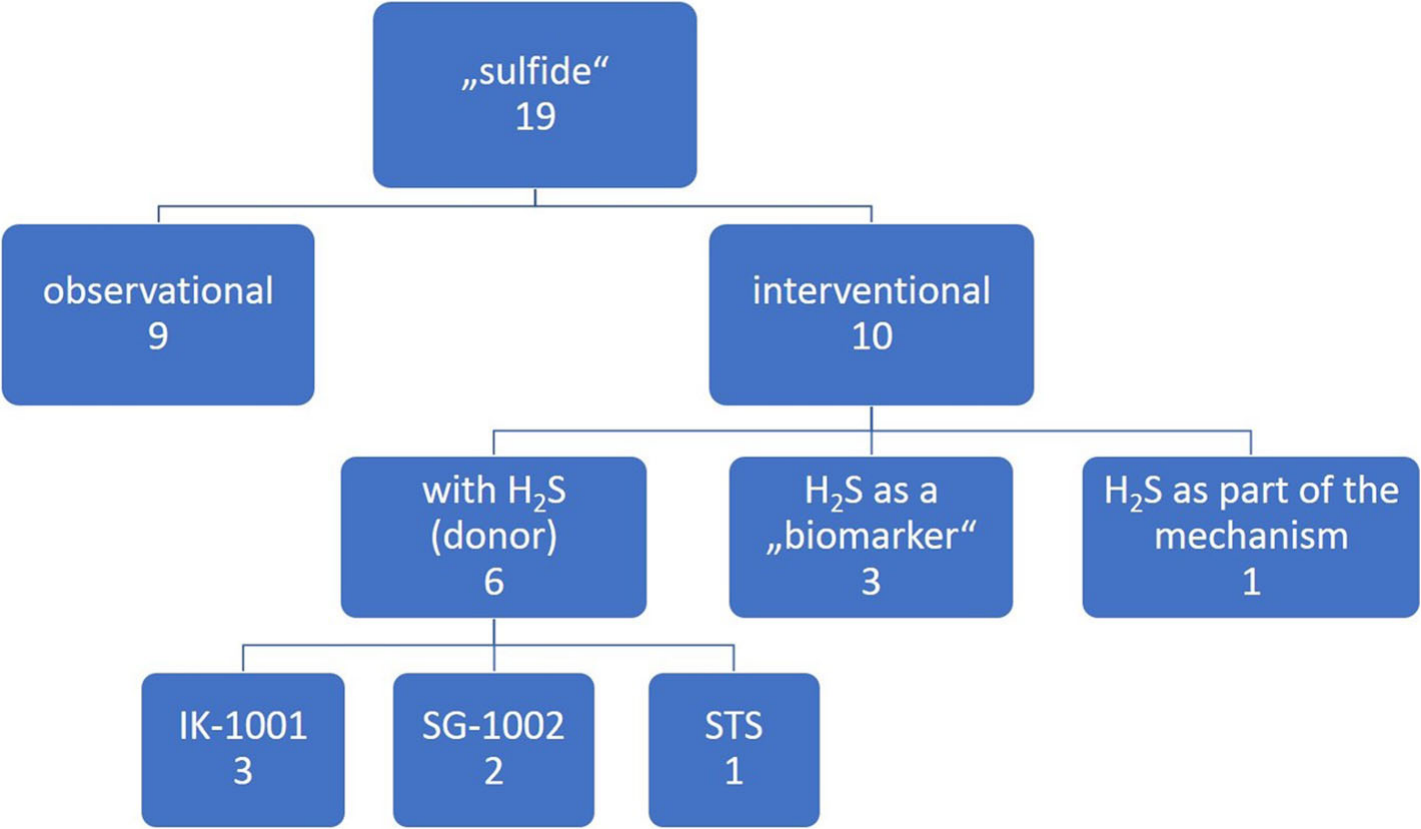
Clinical trials found with the search term “sulfide” on clinicaltrials.gov and relevant to intensive care. Excluded: skin diseases, halitosis, dental conditions, colonoscopy, and similar

To be able to answer the question posted in the title of this review, the clinical development of H_2_S-releasing compounds has to be taken into consideration as well. As we shift from large animal preclinical studies to clinical trials, a search on *clinicaltrials.gov* (August 2019) for the term “sulfide” revealed a total of 64 clinical trials (see Fig. 1). Only two trials were found, which focused on a lung pathology (i.e., asthma), falling into the category “observational” in Fig. 1, investigating the potential use of H_2_S as a biomarker. There are no interventional clinical trials addressing the therapeutic potential of exogenous H_2_S in lung injury or lung disease. Of the 50 interventional trials identified, only 20 were evaluating H_2_S donors, 8 evaluated their intervention based on H_2_S as a biomarker, and 5 suggested H_2_S as a part of the mechanism of their intervention (see Fig. 1). The category “other” in Fig. 1 includes contrast agents, chemotherapeutics, and dietary supplements with a sulfide moiety. Only 6 of the 20 interventional trials with H_2_S donors are relevant to intensive care (see Fig. 2), excluding skin diseases, colonoscopy, and arthritis.

IK-1001, a solution generated by bubbling H_2_S gas into an aqueous solution, was the first compound, designated to administer H_2_S, under investigation in clinical trials in 2009. The first trial of IK-1001 targeted “renal impairment” (NCT00879645) and was terminated prematurely (actual recruitment of 28 participants) because investigators were unable to determine sulfide levels. The issue of not being able to reliably measure sulfide is of course critical for clinical approval of a compound: how would one ever be able to determine the safety of a compound that cannot be measured? One complexity is the fact that exogenous sulfide is highly volatile and rapidly bound and/or metabolized in vivo [27]. Various sulfide pools are available in biological systems and sulfide engages in many different chemical reactions [123], suggesting that these endogenous pools are highly dynamic. Exogenous administration of H_2_S might change the balance of this whole system in ways that we do not fully understand yet. The second trial with IK-1001 in coronary artery bypass (NCT00858936) was terminated after recruiting 6 participants with reasons not reported. The third trial in ST-elevation myocardial infarct (STEMI, NCT01007461) was withdrawn by company decision—non-safety related.

As mentioned above, IK-1001 is an aqueous solution of physically dissolved H_2_S, thus resembling the characteristics of the administration of H_2_S-releasing salts or inhaled H_2_S (see also Table 1). Neither administration of H_2_S via inhalation nor injection of H_2_S-releasing salts will likely be ever used in clinical practice, due to airway mucosal damage and the potential of toxic peak sulfide concentrations, respectively [27]. In fact, inhalation of 300 ppm H_2_S, though sub-lethal, is used as a model to study lung injury [124, 125]. Efforts to avoid the airway irritation of gaseous H_2_S using extracorporeal membrane lung ventilation in a preclinical study were successful, but there was no improvement on the outcome from cardiopulmonary bypass [126].

SG-1002, a mixture of organic sulfide-releasing compounds and salts, has been under investigation in heart failure. A phase I trial revealed the compound to be safe and well tolerated (NCT01989208); a follow-up phase II trial is still in progress with no results posted yet (NCT02278276).

An interesting perspective for H_2_S-based therapeutics is the reconsideration of compounds that are already clinically approved and have only recently been identified to be able to release H_2_S: (i) sodium thiosulfate (STS) [17, 127], approved for cyanide detoxification and cisplatin overdosage; (ii) ammonium tetrathiomolybdate (ATTM) [128, 129], approved for Wilson’s disease, a copper metabolism disorder; and (iii) zofenopril [130], an inhibitor of angiotensin converting enzyme approved for hypertension. These compounds all have been tested extensively and are known to have good safety profiles (see also Table 1).

For example, Dyson et al. showed ATTM led to a 50% reduction of infarct size in rat models of myocardial and cerebral I/R as well as improved survival after hemorrhagic shock [129]. The good safety profile of STS [131] in particular might be related to the fact that thiosulfate itself is an endogenous intermediate of oxidative H_2_S metabolism [127] and is suggested to be “a circulating ‘carrier’ molecule of beneficial effects of H_2_S” [132], in particular under hypoxic conditions [127]. The clinical trial of IK-1001 in renal impairment even used thiosulfate as an indirect measure of H_2_S release from their compound (NCT00879645), although ultimately not successful. STS is currently under investigation in a phase 2 clinical trial to preserve cardiac function in STEMI (NCT02899364). With regard to the lung, as mentioned previously, STS was beneficial in murine models of intratracheal LPS and CLP [72]. Our own group’s findings support these results from Sakaguchi et al.: we determined a beneficial effect of STS to the lung, i.e., improved gas exchange and lung mechanics in a translationally relevant large animal model of hemorrhagic shock (Table 2). Thus, STS is a very promising compound for the development of therapeutic H_2_S administration in ALI in a clinical setting.

## Conclusions

Exogenous H_2_S administration has been demonstrated to be beneficial in various preclinical models of lung injury. However, due to the narrow therapeutic window and width, and potentially toxic effects, the route, dosing, and timing of administration all have to be balanced out very carefully. The development of methods to determine H_2_S levels and/or the pharmacokinetics and pharmacodynamics of H_2_S-releasing compounds is absolutely necessary to facilitate the safety of H_2_S-based therapies. Awaiting the results of currently ongoing clinical trials and the re-evaluation of already approved H_2_S-releasing compounds for novel indications could likely help to prove that H_2_S is in fact not a therapeutic dead end [6].

## Data Availability

The datasets used and/or analyzed during the current study are available from the corresponding author on reasonable request.

## Abbreviations

ALI: Acute lung injury
ARDS: Acute respiratory distress syndrome
ATTM: Ammonium tetrathiomolybdate
CBS: Cystathionine-β-synthase
CLP: Cecal ligation and puncture
CSE: Cystathionine-γ-lyase
DATS: Diallyl-trisulfide
H_2_S: Hydrogen sulfide
ICU: Intensive care unit
I/R: Ischemia reperfusion
i.p.: Intraperitoneal
i.v.: Intravenous
LPS: Lipopolysaccharide
MAP: Mean arterial pressure
MST: 3-mercaptopyruvate-sulfurtransferase
OA: Oleic acid
ppm: parts per million
STS: Sodium thiosulfate
VILI: Ventilator-induced lung injury
